# Multiple-exciton generation in lead selenide nanorod solar cells with external quantum efficiencies exceeding 120%

**DOI:** 10.1038/ncomms9259

**Published:** 2015-09-28

**Authors:** Nathaniel J. L. K. Davis, Marcus L. Böhm, Maxim Tabachnyk, Florencia Wisnivesky-Rocca-Rivarola, Tom C. Jellicoe, Caterina Ducati, Bruno Ehrler, Neil C. Greenham

**Affiliations:** 1 Cavendish Laboratory, University of Cambridge, J.J. Thomson Avenue, Cambridge CB3 0HE, UK; 2 Department of Materials Science and Metallurgy, University of Cambridge, 27 Charles Babbage Road, Cambridge CB3 0FS, UK; 3 Center for Nanophotonics, FOM Institute AMOLF, Science Park 104, 1098 XG Amsterdam, The Netherlands

## Abstract

Multiple-exciton generation—a process in which multiple charge-carrier pairs are generated from a single optical excitation—is a promising way to improve the photocurrent in photovoltaic devices and offers the potential to break the Shockley–Queisser limit. One-dimensional nanostructures, for example nanorods, have been shown spectroscopically to display increased multiple exciton generation efficiencies compared with their zero-dimensional analogues. Here we present solar cells fabricated from PbSe nanorods of three different bandgaps. All three devices showed external quantum efficiencies exceeding 100% and we report a maximum external quantum efficiency of 122% for cells consisting of the smallest bandgap nanorods. We estimate internal quantum efficiencies to exceed 150% at relatively low energies compared with other multiple exciton generation systems, and this demonstrates the potential for substantial improvements in device performance due to multiple exciton generation.

## Abstract (web summary)

One-dimensional nanostructures have been shown to increase multiple-exciton generation, offering a pathway to breaking the Shockley–Queisser limit. Here, Davis *et al*. have fabricated working photovoltaic devices based on high-quality PbSe nanorods and a maximum external quantum efficiency of 122 % was demonstrated.

## Graphical abstract

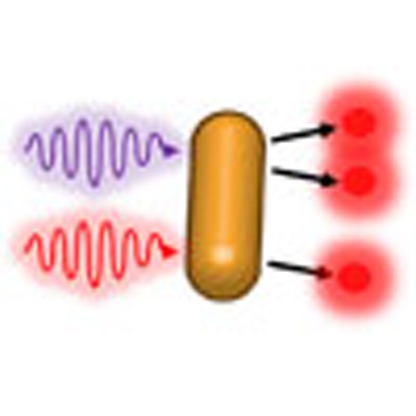

Solar cells fabricated from conventional bulk semiconductors such as silicon or gallium arsenide are approaching the physical limit of solar power conversion efficiency^1^,^2^. Thermalization of hot carriers is the predominant cause of this limitation^2^. A promising strategy to overcome such phonon-related loss processes is to harvest multiple charge-carrier pairs generated from a single excitation. Recently, it has been demonstrated that these mechanisms are particularly efficient in colloidal quantum dots (QDs) where the process is termed multiple-exciton generation (MEG)^3^,^4^,^5^. MEG is enhanced in systems where the limited spatial extent of the excited states (a) relaxes the requirements for conservation of crystal momentum that apply in typical bulk systems^6^ and (b) increases the MEG yield^7^. In an ideal system, bi-exciton states will be formed efficiently once the excitation energy exceeds twice the bandgap.

The multiple-exciton state formed by MEG in PbSe QDs has been shown in spectroscopic experiments to relax on a time scale of 20–200 ps (ref. 3) to a single-exciton state, by an Auger-like process that is the reverse of the MEG process. To harvest charge carriers from multiple-exciton states, it is necessary for charge separation to occur on much faster time scales than Auger decay, and this is not necessarily easy to achieve in a device structure. We note that it is important to achieve two rapid charge transfer events for each doubly excited nanoparticle, since the trion state formed after the first charge transfer event is known to decay rapidly^8^.

Recently, it has been shown that the initial yield of multi-exciton states is enhanced in PbSe nanorod (NR) systems^9^,^10^,^11^. It has been proposed^9^ that this may be due to enhanced MEG rates arising from larger Coulombic electron-hole binding in NRs^12^. Furthermore, Auger relaxation is found to be slowed in these one-dimensional systems^9^. It has been argued that this is due to a slower bimolecular Auger-type recombination in elongated nanostructures compared with a faster, three-particle Auger-type process in zero-dimensional QDs^9^,^13^. NR films are therefore attractive for photovoltaics exploiting MEG; however, fabrication of working devices from NRs has so far proved very challenging^14^.

Here we present the synthesis and characterization of PbSe NRs and their incorporation into working devices. We demonstrate that charges generated by MEG can be extracted from solar cells consisting purely of PbSe NRs with external quantum efficiencies (EQEs) exceeding 120%.

## Results

### Nanorod synthesis

**Figure 1:**
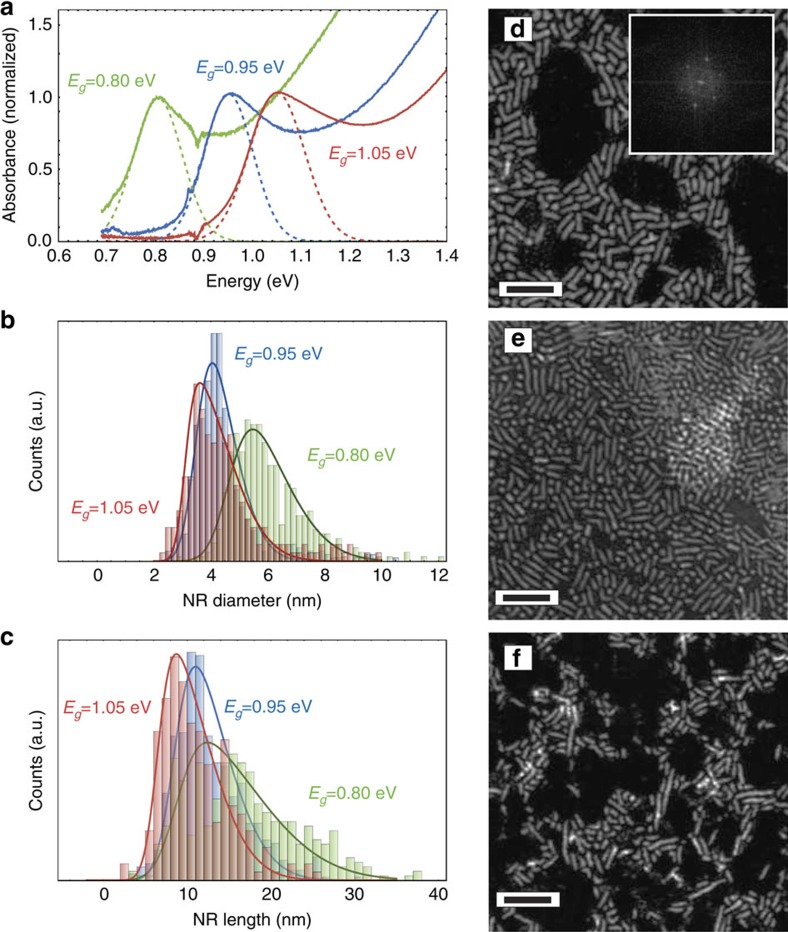
Analysis of synthesized NRs. (**a**) Normalized absorbance spectra of three different CdCl_2_-treated PbSe NR samples in solution. The feature at 0.88 eV is an artefact due to the detector change during the absorbance measurement. (**b**) Short- and (**c**) long-axis distribution of the same bandgap PbSe NRs as determined by scanning transmission electron microscopy (TEM). High-angle annular dark-field (HAADF) TEM images for 0.80, 0.95 and 1.05 eV bandgap samples are shown in **d**–**f**. To confirm the lattice parameters of PbSe in the synthesized NRs, we extract an FFT image from a high-resolution TEM for the 0.8 eV bandgap sample (see inset in **d**). Further TEM and XPS analysis and optimatization details are provided in the Supplementary Figs 1, 2 and 3, respectively. Scale bars, 25 nm (**d**–**f**).

PbSe NRs of three different bandgaps (1.05, 0.95 and 0.80 eV) were synthesized following a method modified from that reported by Koh *et al*.^15^ (see Fig. 1a). We employed an additional *in situ* CdCl_2_ treatment at the end of the NR synthesis to provide additional surface passivation (see the Methods section for further details). It has been shown that this approach minimizes the occurrence of sub-bandgap tail states which improves solar cell performance significantly^16^,^17^. The NR synthesis was optimized to minimize the diameter and length distributions and to reduce the formation of dots and hooks, to improve charge transport^18^,^19^,^20^ (see the Supplementary Note 1). Transmission electron microscopy (TEM) confirmed only negligible quantities (<5% by particle number) of non-NR structures, and diameter and length s.d. of ca. 8% and 13%, respectively (Fig. 1b–f).

### Photovoltaic device fabrication

**Figure 2:**
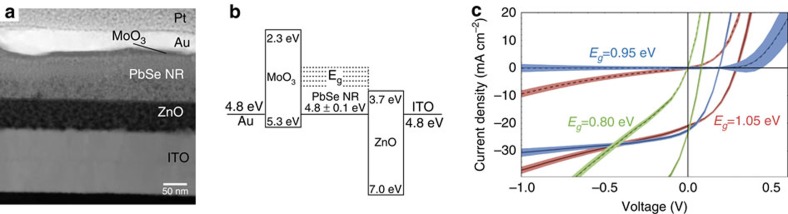
PbSe NR photovoltaic devices. (**a**) Cross-sectional TEM outlining the device architecture (see Supplementary Fig. 6 for details on compositional analysis) and (**b**) energy alignment as determined by a combination of ultraviolet photoelectron spectroscopy (UPS) and absorbance spectroscopy. (**c**) *I*–*V* characteristics of depleted heterojunction solar cells consisting of PbSe NRs with bandgaps 0.80 eV (green), 0.95 eV (blue) and 1.05 eV (red). The dark currents are shown as dashed lines and the *I*–*V* curves under illumination are shown as solid lines. We show the averaged performance of multiple independent solar cells (six cells for 1.05 eV, five cells for 0.95 eV and five cells for 0.80 eV) in dark lines and the spread as a shaded area around the mean.

We fabricated solar cells by depositing a dense array of PbSe NRs on a ZnO film, which was produced using a sol–gel method^5^,^21^ (see Fig. 2a for the device architecture). The NRs were deposited in a layer-by-layer approach using the ligand 1,2-ethaneditiol (EDT) for the first layers and hydrazine as the exchanging ligand for the final NR layer (see the Methods section for further experimental details). It has been shown recently that QDs treated with amine-functionalized ligands exhibit highest occupied molecular orbital (HOMO) and lowest unoccupied molecular orbital (LUMO) levels, which are closer to the vacuum level compared with analogous QD films employing thiol-functionalized ligands^22^. A multi-layer QD film where the bottom layers are treated with EDT and the top layer with hydrazine is therefore likely to show an energy cascading structure that promotes charge extraction^23^. The relevant energy levels were determined using a combination of ultraviolet photoelectron spectroscopy (UPS) and absorbance spectroscopy as described in previous work^24^ (Supplementary Fig. 4) and are presented in Fig. 2b. Current–voltage characteristics of the optimized solar cells made from three different bandgap NRs are shown in Fig. 2c and the standard photovoltaic device parameters are listed in Table 1.

The influence on device performance of choice of ligands, metal oxides and NR synthesis is presented in Supplementary Fig. 5, while the optimized device architecture is shown in Fig. 2a. We attribute the non-ideal diode behaviour seen particularly for the 0.80 eV NR device to tail states in the sol–gel processed ZnO (ref. 25) and remaining trap states in the PbSe NR film^24^. These tail states allow trap-induced leakage current especially in devices fabricated from small-bandgap NRs under reverse bias, thereby reducing the quality of the diode in the dark. Under illumination, we expect these tail states to promote trap-assisted recombination, thereby reducing the open-circuit voltage^24^,^26^.

### Device quantum efficiencies

**Figure 3:**
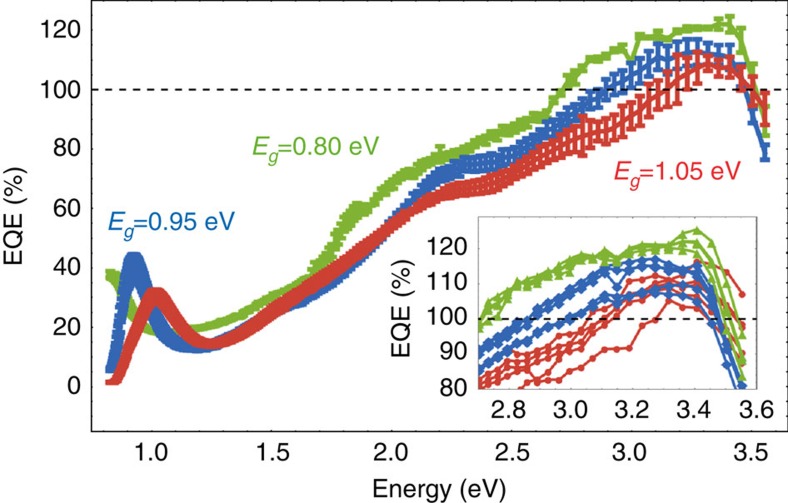
External quantum efficiencies (EQE) of PbSe NR photovoltaic devices. The bandgaps of the NRs used are 0.80 eV (green), 0.95 eV (blue) and 1.05 eV (red). The inset shows the high-energy region of the individual EQE spectra of the solar cells displaying quantum efficiencies >100%. Error bars show the standard deviation of multiple independent solar cells (six cells for 1.05 eV, five cells for 0.95 eV and five cells for 0.80 eV).

Figure 3 displays the short-circuit EQE spectrum for NR devices. Interestingly, we observe maximum EQEs of 109±3%, 113±4% and 122±3% for devices with NR bandgaps of 1.05, 0.95 and 0.80 eV, respectively, at high-photon energies (ca. 3.3 eV). We note that no antireflective coating was employed to reduce reflectance losses at the glass/air interface. Reassuringly, we can reconstruct the measured short-circuit current within ca. 1% measurement error by integrating the EQE over the AM1.5G solar spectrum (Supplementary Table 1). Furthermore, measuring the EQE under different white light biases produced identical spectra, suggesting a current collection that is independent of the charge-carrier density (Supplementary Fig. 7). For the lowest photon energies, we recognize a clearly visible first excitonic peak in all three test devices and explain the steep drop in quantum efficiency for photon energies exceeding 3.5 eV by the onset of absorption of the ZnO layer (Supplementary Fig. 8).

**Figure 4:**
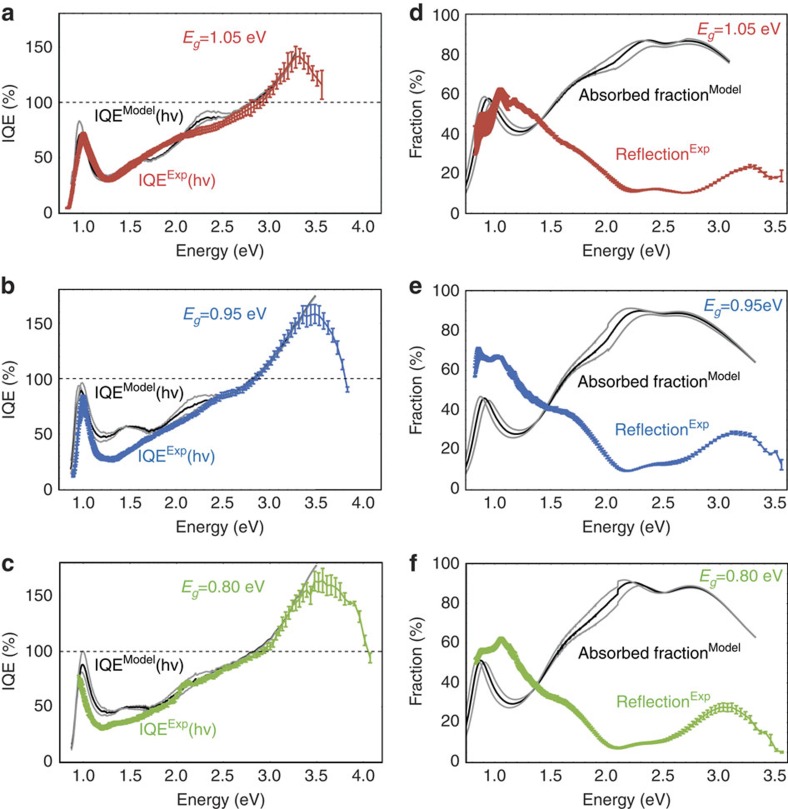
Internal quantum efficiency (IQE) of photovoltaic devices. NRs with bandgaps of 1.05 eV (**a**), 0.95 eV (**b**) and 0.80 eV (**c**). IQE^EXP^(*hv*) and IQE^Model^(*hv*) were determined, respectively, using reflectance measurements and optical modelling as described in the text. (**d**–**f**) The measured reflection (coloured curve) and modelled absorbed fraction for bandgaps of 1.05, 0.95 and 0.80 eV, respectively. Error bars show the standard deviation of multiple independent solar cells (six cells for 1.05 eV, five cells for 0.95 eV and five cells for 0.80 eV). The range of grey curves shown for the modelled results illustrate the effect of changing the active layer thickness in the model by the experimental error of ±10 nm.

To allow for incomplete absorption of incident photons, we next determined the internal quantum efficiency (IQE) via two independent approaches: first, we measure the fraction of light reflected from the device at each photon energy *R*(*hυ*) using a calibrated silicon or germanium photodiode. The IQE was then calculated as 
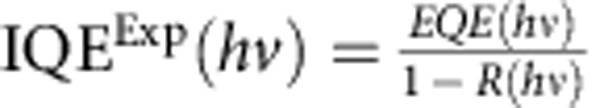
. We note that IQE^Exp^ (*hv*) presents the lower bound for the IQE, as parasitic absorbance by other layers and diffuse scattering are negelected^5^. In our second approach, we derive IQE^Model^(*hv*) by applying an optical transfer matrix model^27^,^28^ using the refractive indices *n* and the extinction coefficients *k* of each device layer measured by ellipsometry (Supplementary Fig. 9). In this case, 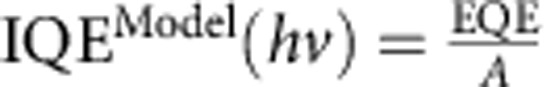, where *A* is the calculated fraction of light absorbed. Reassuringly, we identify similar values for IQE^Exp^(*hv*) and IQE^Model^(*hv*) (Fig. 4). These values at their highest are above 170%, which is comparable to devices incorporating singlet fission materials to generate multiple excitons^28^,^29^,^30^. We note that the dip in EQE after the first excitonic absorption peak is deeper than would be expected based on the absorption spectrum (Supplementary Fig. 10), leading to a significant dip in the calculated IQE spectrum in the same spectral region. This phenomenon is difficult to explain, but is seen in many nanocrystal devices^5^,^31^,^32^,^33^. It is unlikely to result from charge generation taking place deeper in the device when the absorption coefficient is lower, as these effects should recover in full when the absorption coefficient regains its initial peak value at around 1.4 *E*_g_ (see Supplementary Fig. 10 for film absorption).

**Figure 5:**
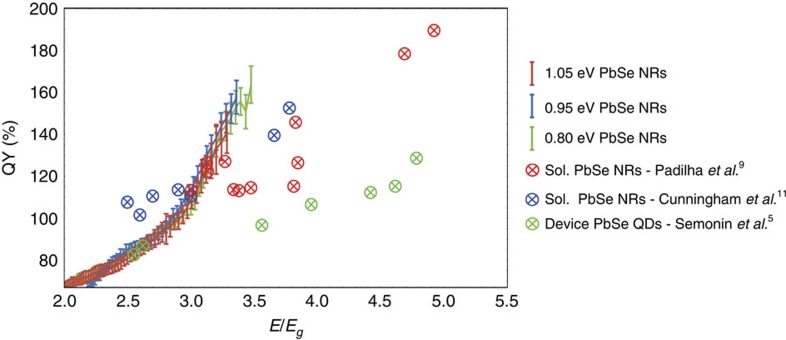
Comparison IQEs with literature. IQEs of devices consisting of PbSe NRs with bandgaps of 1.05, 0.95 and 0.80 eV and MEG quantum yields of, PbSe QDs in devices^5^ and PbSe NRs in solution^9^,^11^. Error bars show the standard deviation of multiple independent solar cells (six cells for 1.05 eV, five cells for 0.95 eV and five cells for 0.80 eV). A comparison including MEG quantum yields of PbSe QDs in solution^34^,^36^ and PbSe QDs in films^35^ can be found in Supplementary Figure 11.

In Fig. 5, we show the IQE above 2*E*_*g*_ for all three nanoparticle sizes as a function of energy normalized to the respective bandgap energy. An ideal MEG system would show sharp increases in quantum efficiency at multiples of the bandgap. However, in common with other reports^5^,^9^,^10^,^34^,^35^,^36^, we find a gradual increase in efficiency above 2*E*_*g*_. The threshold energy at which this increase begins, and the rate of efficiency increase above the threshold are important parameters in comparing materials systems and in determining the gain in power conversion efficiency due to MEG for a device under solar illumination. Beard *et al*.^4^ have considered a model in which the rate of MEG increases with energy above threshold, leading to a gradual rise in initial MEG yield as this process competes with rapid cooling. In a device, quantum efficiency enhancement depends not only on the initial yield of multiple excitons, but also on being able to rapidly separate and efficiently collect the additional charge carriers. From our data, we make the following observations: the dependence of IQE on bandgap-normalized energy is remarkably similar for all three NR bandgaps, exceeding 100% at around 2.9*E*_*g*_ in all cases and reaching 150% by 3.4*E*_*g*_. This is a substantial improvement over the dot devices reported by Semonin *et al*.^5^, where the IQE increases much more slowly with energy, not reaching 150% until nearly 5*E*_*g*_. In our data, it is difficult to accurately determine a threshold energy for MEG, due to the energy-dependent quantum efficiency below 2*E*_*g*_ that is discussed above. Clearly quantum efficiency without the assistance of MEG cannot be >100%, so the MEG threshold must be below 2.9*E*_*g*_. Taking a quantum efficiency of 75–80% with no MEG contribution, consistent with the IQE values at the first excitonic peak, suggests an MEG threshold below 2.5*E*_*g*_, and if the energy dependence of IQE in Fig. 5 were solely due to MEG then the threshold would be close to 2*E*_*g*_. It is interesting to compare the IQE energy dependence with spectroscopic measurements of initial MEG yields in PbSe dots^34^,^36^ and rods^9^,^11^ in solution, also shown in Fig. 5 and in the Supplementary Fig. 11. Despite the fact that device IQEs are reduced by Auger recombination competing with charge separation, and by regular recombination losses, the IQEs we measure increase more rapidly with energy than the initial MEG yields in solution. This indicates that MEG is enhanced in films, an important result when attempting to make predictions about MEG in devices based on spectroscopic measurements in solution. This enhancement of MEG in films has been noted by Sandeep *et al*.^35^, who use microwave conductivity measurements to determine MEG yields at longer times in films of PbSe dots. In those measurements, the threshold was close to 2*E*_*g*_, but the yield of additional carriers was strongly dependent on the choice of ligands, with quantum efficiencies comparable with ours seen only in films with the shortest, 1,2-ethanediamine, ligands, presumably due to the short ligands allowing rapid charge separation between nanoparticles. The reason for such a low MEG threshold energy remains unclear; mechanisms proposed include the formation of interparticle band structure in the solid state^35^ or a trap-assisted MEG mechanism^37^, Interestingly, Sandeep *et al*.^35^ observed inefficient charge-carrier generation from MEG in PbSe dot films using the same 1,2-ethanedithiol ligand that we use here (Supplementary Fig. 11). The difference may be due to the change from dots to rods, or due to the additional hydrazine treatment that we apply. Our measurements on NR devices demonstrate that carriers from MEG can not only be separated locally to contribute to microwave conductivity, but can also be collected efficiently in a solar cell structure.

Finally, we estimate the contribution of MEG to the photocurrent in our devices under solar illumination. To do this, we make the (very conservative) assumption that only the fraction of the IQE in excess of 100% is due to MEG, and we weight the measured EQE by that fraction before integrating over the solar spectrum (Supplementary Fig. 12). We find that the short-circuit current under AM1.5G illumination (Supplementary Table 2) is enhanced by at least 1.7%, 4.5% and 5.8% for NR bandgaps of 1.05, 0.95 and 0.8 eV, respectively. Assuming a more realistic quantum efficiency without MEG of 80% leads to enhancements as high as 12.5% for the 0.8 eV sample, compared with the 4% enhancement estimated by similar methods for dot devices^5^. MEG thus contributes a substantive amount to the device efficiency, in contrast to the effects seen in bulk semiconductors such as Si_1−*x*_Ge_*x*_ alloys where carrier multiplication effects increase the photocurrent by at most 2%.

## Conclusion

We have demonstrated working photovoltaic devices based on high-quality CdCl_2_-treated PbSe NRs of three different bandgaps. EQE values clearly exceeded 100 %, and maximum EQEs of 122% were found for the smallest bandgap devices. Estimated IQE values were found to increase rapidly above 2*E*_*g*_, reaching values as high as 170% at only 3.5*E*_*g*_. This behaviour is superior to that seen in solution-based measurements of MEG yields, and indicates potential for substantial efficiency gains in MEG-based solar cells.

## Methods

### PbSe nanorod synthesis

The synthesis of PbSe NRs was carried out following modified versions of previously reported methods (see Supplementary Note 1)^15^.

Briefly, PbO (1.76 g, 7.8 mmol), oleic acid (6.2 ml, 19.7 mmol, 5.6 g) and octadecene (41.8 ml, 127.6 mmol, 32.6 g) were combined in a three-neck flask and degassed at 110 °C under vacuum (10^−2^ mbar or better) for 2 h. Subsequently, the reaction flask was flushed with nitrogen and heated to 160 °C. In parallel, CdCl_2_ (0.16 g, 0.9 mmol), tetradecylphosphonic acid (33 mg, 0.12 mmol) and oleylamine (8.13 ml, 30.4 mmol, 8.1 g) were combined in a separate three-neck flask and degassed under vacuum (10^−2^ mbar or better) at 110 °C for 12 h. The solution was flushed with nitrogen and set to 100 °C. A solution of selenium (1.92 g, 23.8 mmol) in tris(diethylamino)phosphine (TDP; 24.0 ml, 87.6 mmol; 20.8 g) was rapidly injected into the lead precursor solution. The bandgap of the PbSe NRs was tuned by adjusting the reaction temperature while the overall reaction time was kept constant at 2.5 min. For bandgaps of 1.05, 0.95 and 0.80 eV, reaction temperatures of 120, 130 and 140 °C were chosen, respectively. For the *in situ* CdCl_2_ treatment, 2.7 ml of the CdCl_2_/tetradecylphosphonic acid solution was injected into the reaction flask of the NRs, 10 s before the crystal growth was quenched. The reaction was quenched by adding 20 ml of ice-cold *n*-hexane and by placing the reaction flask in an ice-water bath. The NRs were isolated from the reaction mixture by flocculating to turbidity using a 1-buthanol/ethanol/hexane solvent system. The purified QDs were then redispersed in octane at a concentration of ∼100 mg ml^−l^ and stored under argon.

### Nanorod analysis

Absorption spectra in solutions were measured on NR samples dispersed in tetrachloroethylene at a concentration of ca. 1 mg ml^−1^ using a PerkinElmer Lambda 9 ultraviolet–visible–infrared spectrometer. Film absorption spectra were taken from PbSe NRs, which were prepared on quartz glass using a modified version of a literature reported layer-by-layer deposition method^38^. Briefly, PbSe NRs were spin coated on the substrate at a concentration of 25 mg ml^−1^ in octane (1,500 r.p.m. for 15 s) after a wait of 5 s. Subsequently, the native oleic acid ligand was exchanged with ethane dithiol (20 mmol in acetonitrile) in a second spin-coating step using the same spinning conditions. To remove residual ligand and un-exchanged NRs consecutive spin-rinsing steps using pure acetonitrile and octane were performed. This cycle was repeated four times. For the final NR layer, hydrazine (1 M in acetonitrile) instead of EDT as the exchanging ligand. The NR films were encapsulated by affixing a glass coverslip on the NR layer using carbon tape as spacer unit and epoxy glue as sealant. TEM samples were prepared as reported elsewhere by drop casting a ca. 1 mg ml^−1^ NR solution in octane on a TEM Grid (200 Mesh Cu, Agar Scientific) in a nitrogen-filled glove box.

### Photovoltaic device fabrication

Solar cells were prepared on indium tin oxide (ITO)-patterned glass substrates cleaned in an ultrasonic bath with ethanol, acetone and isopropanol. A ca. 55-nm ZnO layer (Supplementary Figure 8) was deposited using a sol–gel method suggested by Lloyd *et al*.^39^ and modified by Beek *et al*.^21^ Briefly, 250 μl of diethylzinc in hexane (1 M) was diluted in 750 μl anhydrous tetrahydrofuran in a nitrogen-filled glove box. The solution is spun-cast in air at 4,000 r.p.m. for 30 s. The ZnO films were then allowed to rest at room temperature under ambient environment for 15 min and were then annealed at 130 °C for 5 min. PbSe NRs were deposited following a sequential layer-by-layer spin-coating technique as described above. The samples were then transferred into a thermal evaporator and molybdenum oxide (MoO_*x*_; 7 nm) and gold (Au; 100 nm) were deposited through a shadow mask at 3 × 10^−6^ mbar or better. The solar cells were encapsulated by affixing a glass slide on top of the contacts using transparent epoxy glue.

### Photovoltaic device characterization

A 100-W tungsten halogen lamp (500–1,500 nm) and a 120-W Xenon lamp (350–500 nm) dispersed through a monochromator (Oriel Cornerstone 260) was used for EQE measurements. For wavelengths between 1,500 and 800 nm, a set of InGaAs detectors were employed, (ThorLabs SM1PD2A) and for wavelengths between 900 and 350 nm a set of silicon diodes (ThorLabs SM05PD1A) were used. A Keithley 2635 source measure unit was used to measure the short-circuit current as a function of wavelength. The incident light was focused to a spot size of ca. 1 mm^2^ using a set of lenses to illuminate the individual pixel of size 5.5 cm^2^. Current–voltage characteristics were measured under AM 1.5G conditions using an Abet Sun 2000 solar simulator, at an intensity equivalent to 100 mW cm^−2^ after correcting for spectral mismatch. Both the dark and light current–voltage characteristics were measured using the Keithley 2635 source measure unit.

### Internal quantum efficiency measurements

IQE is represented as EQE/absorbed light fraction^5^. The absorbed light fraction was found by measuring the reflectivity at ca. normal incidence of a device using a photodiode. A 100-W tungsten halogen lamp (500–1,500 nm) and a 120-W xenon lamp (350–500 nm) dispersed through a monochromator (Oriel Cornerstone 260) was used. For wavelengths between 1,500 and 800 nm, a set of InGaAs detectors, (ThorLabs SM1PD2A) were employed and for wavelengths between 900 and 350 nm a set of silicon diodes (ThorLabs SM05PD1A) were used. The absorbed light fraction is then determined by 1−*R*, where *R* is the reflectivity.

### Photoelectron spectroscopy

For photoelectron spectroscopy 3 nm chromium and 80 nm gold were thermally evaporated onto cleaned silicon substrates. The QDs were deposited in a layer-by-layer fashion as described above. The samples were then transferred into the vacuum chamber of a Thermo Scientific ESCALAB 250Xi X-ray photoelectron spectrometer (XPS) minimizing air exposure (about 10 sec).

UPS measurements were performed using a double-differentially pumped He gas discharge lamp (He I radiation (*hv*=21.2 eV); pass energy 2 eV). In Supplementary Fig. 4, the spectra are presented as a function of binding energy with respect to vacuum level.

XPS was performed using an XR6 monochromated Al K_α_ X-ray source with an energy *hv*=1,486.6 eV and a spot size of 650 μm. To prevent the samples from surface charging an argon-ion flood gun was used. For data analysis of both UPS and XPS spectra, the software package ‘Thermo Avantage' (Thermo Fisher Scientific Inc., Waltham, USA) was used.

### Transmission electron microscopy

Samples were prepared by affixing a TEM grid (200 Mesh Cu, Agar Scientific) onto a glass substrate. A single layer of PbSe QDs was deposited from an octane solution (ca. 5 mg ml^−1^) and was ligand exchanged with the ligand mixture solution following methods described above. The prepared TEM grid was then removed from the glass substrate and imaged employing a FEI Tecnai F20 microscope operated at 200 kV. For high-resolution TEM, the same microscope and conditions were used. TEM analysis of the crystal orientation and lattice spacing is shown in Supplementary Fig. 1.

The preparation of a cross-sectional lamellar specimen was carried out on a FEI Helios dual-beam FEG scanning electron microscopy (SEM)/focused ion beam (FIB) microscope, fitted with an Omniprobe micromanipulator for *in situ* lift-out. The sample preparation was performed following a standard FIB *in situ* lift-out technique^33^, and the thinning step of the lamellar specimen was performed with decreasing beam current to reduce sample damage and improve sputtering of the material. The cross-sectional specimen was analysed through high-angle annular dark-field scanning TEM, using a Fischione detector on a FEI Tecnai F20 microscope, operated at 200 kV.

Energy dispersive X-ray spectroscopy mapping of the cross-sectional specimen was performed using a FEI Tecnai Orisis TEM/scanning TEM equipped with a field-assisted thermionic emitter gun, operating at 200 kV. The microscope is also equipped with four Bruker silicon drift detectors for high collection efficiency and high count rates.

### Ellipsometry

For ellipsometry, all samples were prepared as described above but on silicon substrates. The only exception is ITO, which was measured on glass as received from Psiotec. The samples were measured on a Woollam Vase VB-400 ellipsometer in reflection mode (ITO in transmission mode) using monochromatic light from a xenon lamp guided through a monochromator. The data for the QD samples were fitted using a combination of a Cauchy and a Gaussian model. The ITO data were fitted with a combination of a Drude and a Lorentz oscillator and the MoO_*x*_ was fitted with a Lorentz oscillator.

### Transfer matrix modelling of IQE

Reflectance was modelled as per literature sources^25^,^41^. The *n* and *k* values were measured in-house as described in the ellipsometry section above and are presented in Supplementary Figure 9. This program calculates the field profile, exciton generation profile and generated current from the wavelength dependent complex indices of refraction in devices using a transfer matrix method described in detail in refs 27, 28. It assumes the light source located in an *n*=1 environment (air) and that the first layer is a thick substrate, so that incoherent reflection at the air/1st layer interface is taken into account before the coherent interference is calculated in the remaining layers. Film thicknesses were measured using a DEKTAK profilometer. Error in the model is given as ±10 nm of the active layer.

## Additional information

**How to cite this article**: Davis, N. J. L. K. *et al*. Multiple-exciton generation in lead selenide nanorod solar cells with external quantum efficiencies exceeding 120%. *Nat. Commun*. 6:8259 doi: 10.1038/ncomms9259 (2015).

## Supplementary Material

Supplementary Information Supplementary Figures 1-13, Supplementary Tables 1-2, Supplementary Note 1 and Supplementary References.

## Figures and Tables

**Table 1:** Photovoltaic parameters of PbSe NR champion devices with three different bandgaps.

*E*_*g*_ (eV)	*J*_sc_ (mA cm^−2^)	*V*_oc_ (V)	FF (%)	*η* (%)
1.05	21.0	0.29	41	2.52
0.95	22.6	0.19	37	1.61
0.80	23.5	0.08	28	0.54
