# Supplementary material for: Multiple-exciton generation in lead selenide nanorod solar cells with external quantum efficiencies exceeding 120%
Source: Nat Commun. 2015 Sep 28;6:8259. doi: 10.1038/ncomms9259 (PMC4667436; doi:10.1038/ncomms9259)
Supplement: Supplementary Information — Supplementary Figures 1-13, Supplementary Tables 1-2, Supplementary Note 1 and Supplementary References. [file ncomms9259-s1.pdf]

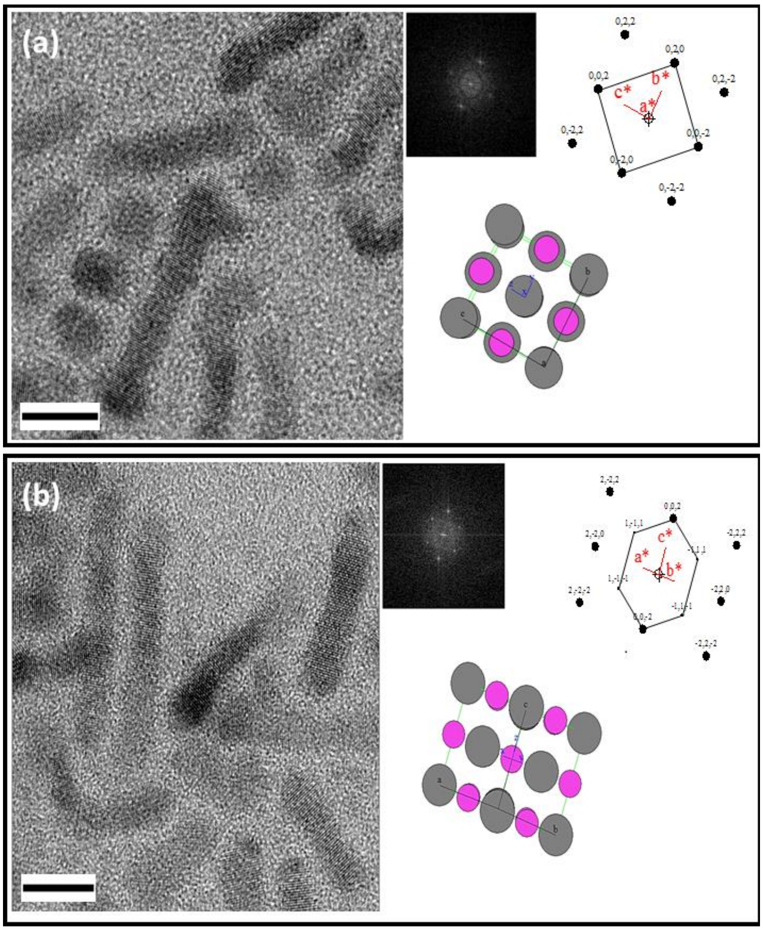

Supplementary Figure 1: Lattice spacing and crystal orientation of PbSe nanorods; (a) Imaged from the PbSe zone Axis [1 0 0] (d1 = 3.06 Å, d2 = 3.06 Å, Angle = 90°). (b) Imaged from the PbSe zone Axis [1 1 0] (d1 = 3.53 Å, d2 = 3.53 Å, Angle = 90°). Scale bars correspond to 10 nm

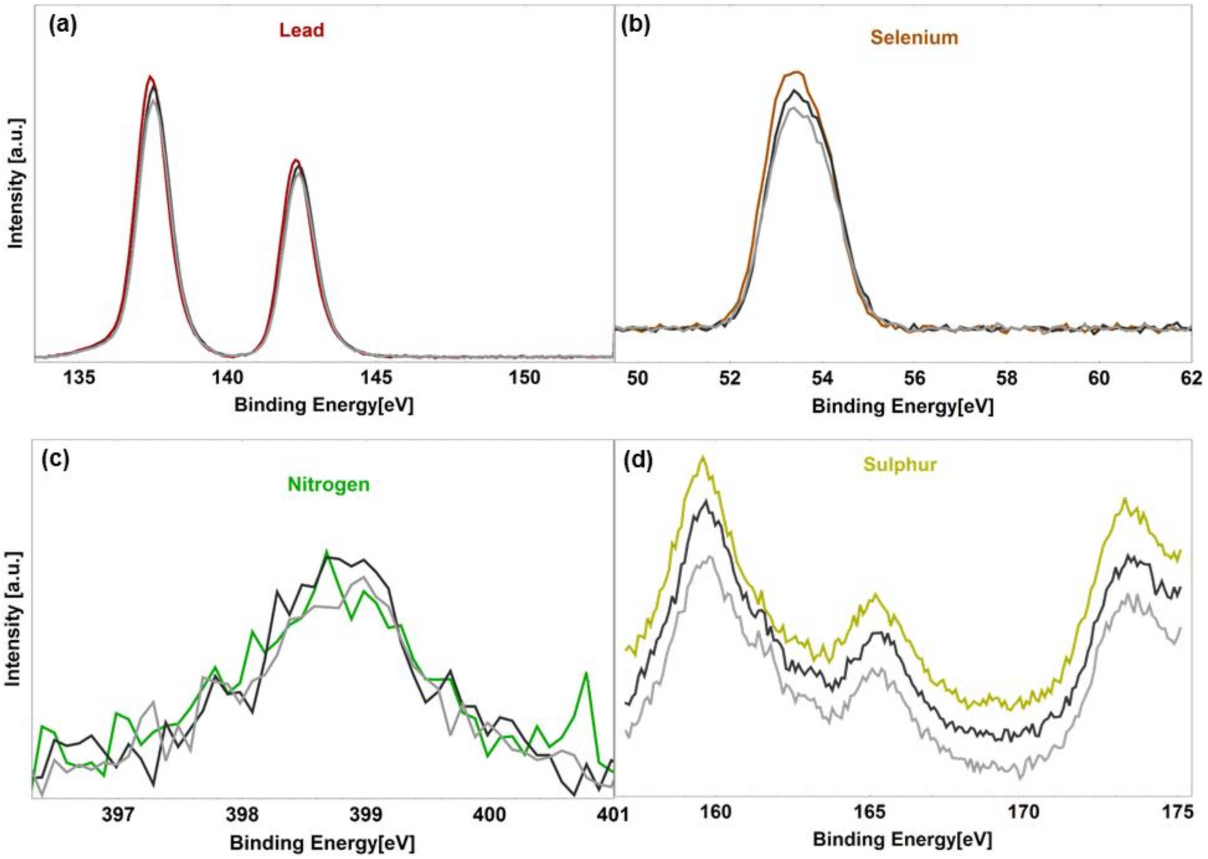

Supplementary Figure 2: X-ray photoelectron spectroscopy conducted on $CdCl_2$-treated PbSe nanorod films. These were prepared using EDT and hydrazine as the final ligand species. Three different bandgap PbSe nanorods were measured: 0.80eV (colour), 0.95eV (black) and 1.05eV (gray). See supplementary Note 1.

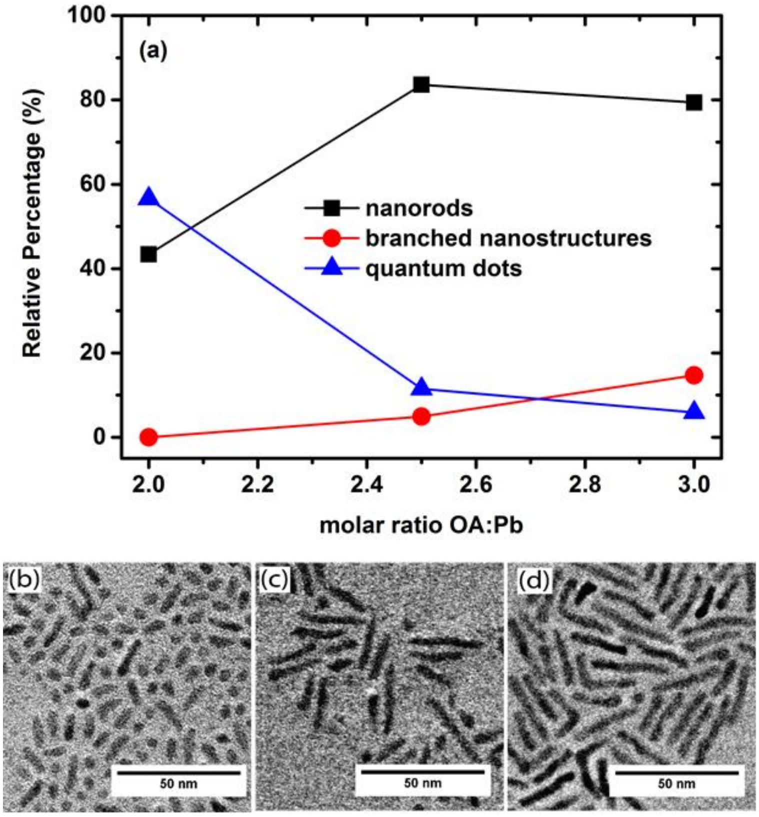

Supplementary Figure 3: (a) Relative percentage of nanostructure species formed as a function of OA:Pb stoichiometric ratio. Transmission electron microscopy (TEM) images of nanostructures formed in a reaction employing a 2.0 (b), 2.5 (c) and 3.0 (d) molar ratio of OA and Pb in the Pb-precursor solution. All TEMs shown are before size selective precipitation to remove residual quantum dots.

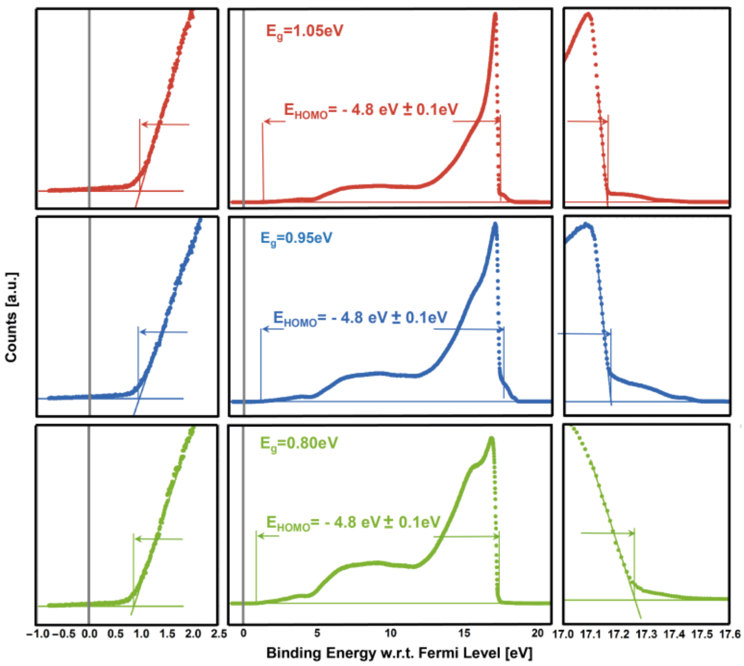

Supplementary Figure 4: Ultraviolet photoelectron spectroscopy (UPS) of films consisting of $CdCl_2$-treated PbSe nanorods. These were deposited in a layer-by-layer approach using EDT and hydrazine as the final ligand species.We identify the LUMO level of the nanorods as demonstrated previously.[2] Briefly, we determine the bandgap of the nanorods from the energy of the first excitonic peak in the solution absorption spectrum (Figure 1 in the main text) and subtract this value from the HOMO level measured by UPS to identify the LUMO level of the nanorods.

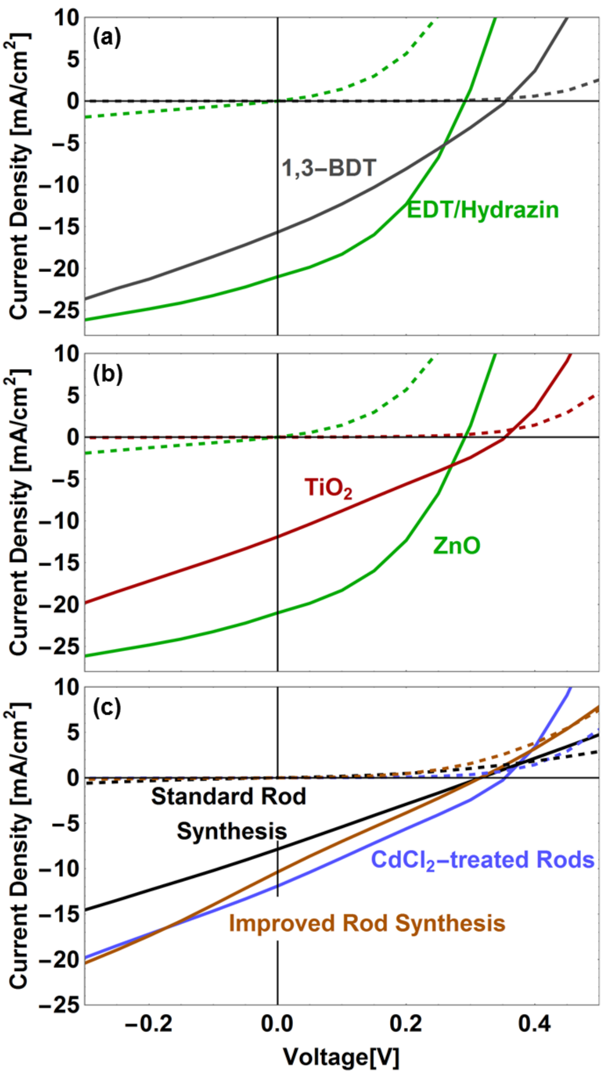

Supplementary Figure 5: Optimization of device parameters. The final surface ligands (a), the metal oxide (b) and the synthesis conditions as outlined in the methods (c) were optimised. In (a) the $CdCl_2$-treated PbSe NRs (E_g=1.05eV) where deposited on ZnO. In (b) we compared the same NRs passivated with EDT and hydrazine on TiO_2 and ZnO. In (c) we correlated the PV performance of differently synthesised NRs (E_g=0.91eV) deposited on ZnO. We compare the performance of NR devices, which were ligated with either 1,3 benzene dithiol (BDT) or the combination of 1,2-ethane dithiol (EDT) and hydrazine. We found that the dual ligand approach with EDT and hydrazine produces higher short circuit currents and fill factors and reduces the open circuit voltage marginally (see (a)). This effect has been explained in literature with an enhanced charge carrier mobility in NC films treated with amine ligands.[3,4] Furthermore, replacing TiO_2 as electron-collecting layer with ZnO showed similar improvements to the photovoltaic parameters (see (b)). We tentatively assign the increased short-circuit current to higher charge mobilities in ZnO compared to TiO_2[5,6] and the mildly reduced open circuit voltage to a greater abundance of sub-bandgap tail states in ZnO.[7] We next study the effect of the improved nanorod synthesis (i.e. suppression of residual 0-diminsional quantum dots and remaining “hook/cross”-nanostructures in the nanorod sample) as well as the applied $CdCl_2$-treatment on the device performance. While the “cleaner” nanorod sample shows mainly an increase in short-circuit current, it is the additional $CdCl_2$-treatment which improves all relevant photovoltaic parameters (i.e. V_{OC}, fill factor as well as the J_{SC}, see (c)). We speculate that the first effect may be associated with an improved NR bulk morphology[8,9] and assign a refined surface passivation to be responsible for the latter improvement.[10]

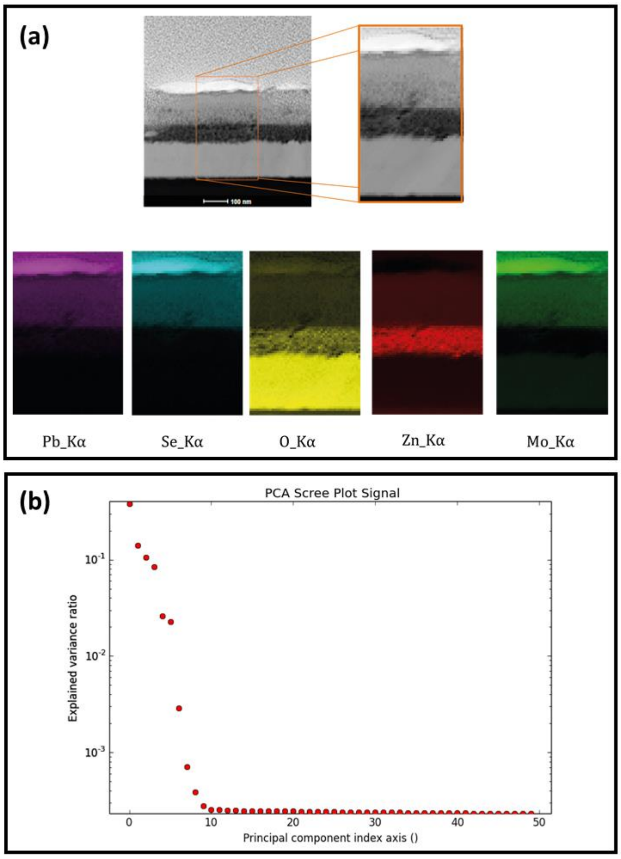

Supplementary Figure 6: (a) EDX Compositional maps. The images were de-noised using principal component analysis (PCA) and the maps were plotted from the resulting intensity profile for each element. (b) Principal component analysis (PCA) has been used to reduce the noise from the acquired EDX spectra. PCA is a multivariate statistical method that sorts the components in the data in order of decreasing variance. In this case it was used to estimate the dimensionality of the data by plotting the explained variance against the component index in a logarithmic y-scale. This plot shows a quick drop, eventually becoming a slowly descending line. The point at which it becomes linear is considered to give an estimate of the number of components within the dataset. For this case, nine (9) components were identified. The de-noising property of PCA is achieved by using a reduced set of components to make a model of the original signal, reducing the dimensionality of the data, and consequently the noise. X-ray line intensities were then extracted from the EDX spectrum from each component, generating the individual intensity maps for the elements of interest. In order to facilitate the interactive data analysis of these complex datasets, HyperSpy an open-source, free software package, has been used to analyse the EDX data from HAADF-STEM spectrum images.

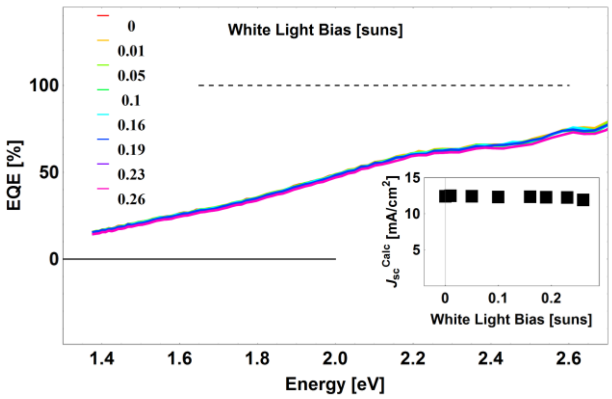

Supplementary Figure 7: White light bias dependent EQE spectra of devices consisting of NRs of the bandgap 1.05 eV. The short-circuit current shown in the inset was calculated by integrating the EQE against the AM1.5G solar spectrum. Due to the lack of photocurrent for excitations of 2.7 eV and higher energies as well as the lack of the photocurrent in the infra-red the calculated values are smaller than the ones listed in Supplementary Supplementary Table **1**. The white light bias was calibrated against the photon flux of an AM1.5G solar simulator using a reference silicon solar cell (Czibula & Grundmann 015-2008).

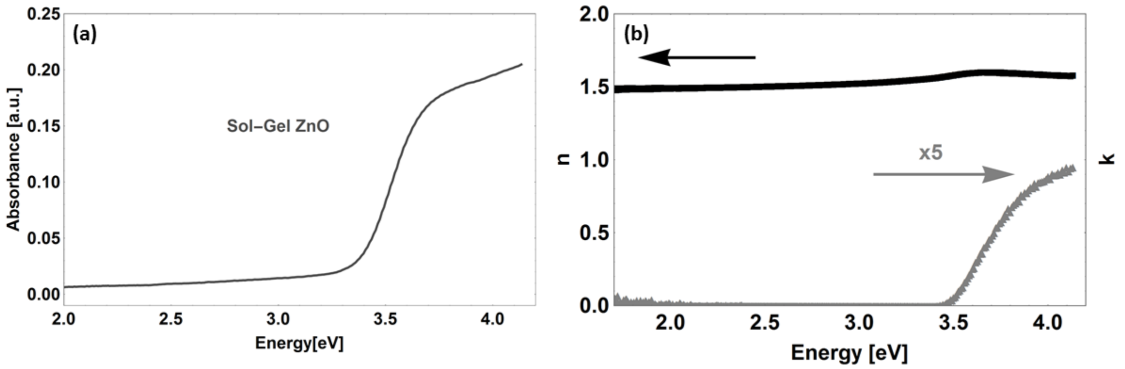

Supplementary Figure 8: Absorption and ellipsometry of Sol-Gel ZnO films. (a) film absorbance of sol-gel processed ZnO. (b) film thickness determination using the refractive index *n* and extinction coefficient *k* determined using ellipsometry. We determine the film thickness of ZnO by ellipsometry. Using the phase delay caused by the interference between the light reflected from the surface and the light which travelled through the film we relate the physical film thickness with the index of refraction *n*. We identify a film thickness of ca. 55nm.

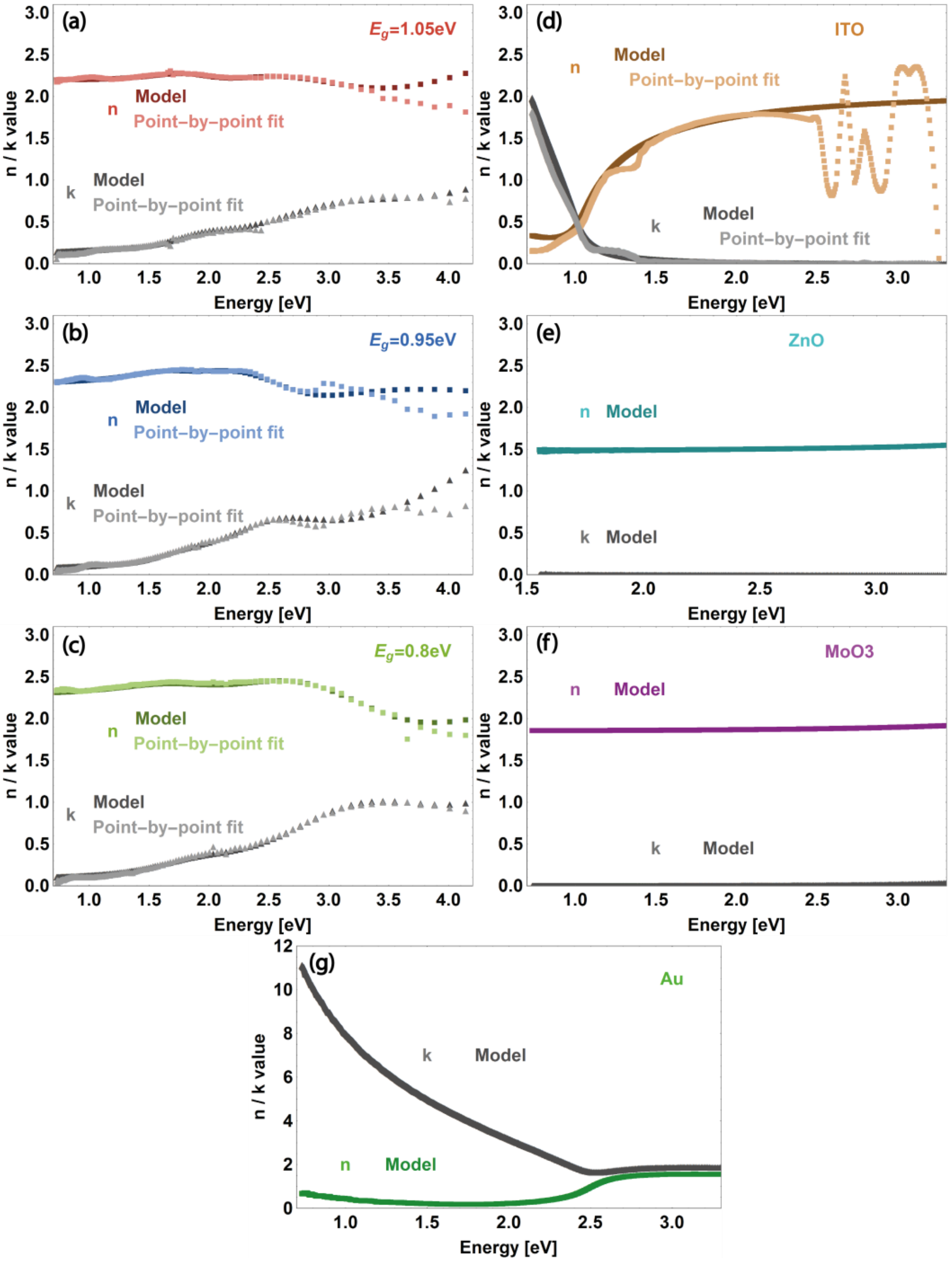

Supplementary Figure 9: Determination of the refractive index *n* and the extinction coefficient *k* of PbSe NRs of (a) 1.05eV E_g, (b) 0.95eV E_g (c) 0.8eV E_g, (d) ITO, (e) ZnO, (f) MoO3 and (g) Au layers using ellipsometry. Layer thickness are as follows: 1.05 eV PbSe rods 115 ± 10 nm, 0.95 eV PbSe rods 124 ± 10 nm. 0.80 eV PbSe rods 122 ± 10 nm, ITO 150 nm, ZnO 55 ± 5 nm, MoO3 8.0 nm ± 0.1 nm, Au 100 nm ± 1 nm.

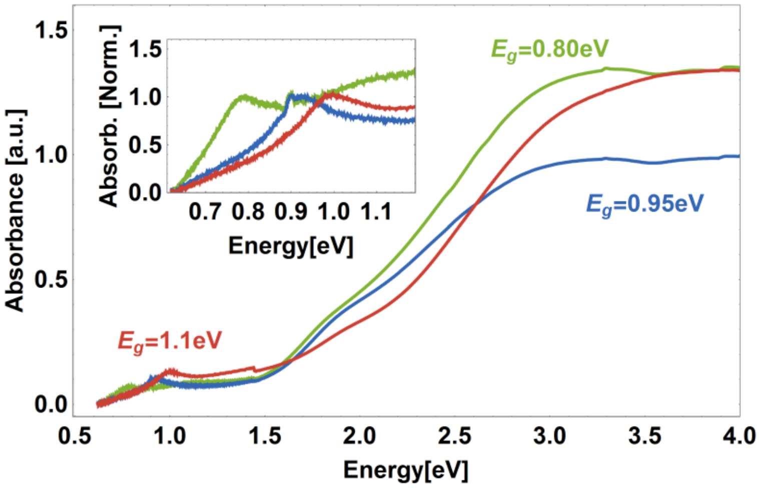

Supplementary Figure 10: Film absorbance spectra of three different bandgap PbSe nanorod samples. The inset shows the normalized absorbance of the region of the first excitonic peaks.

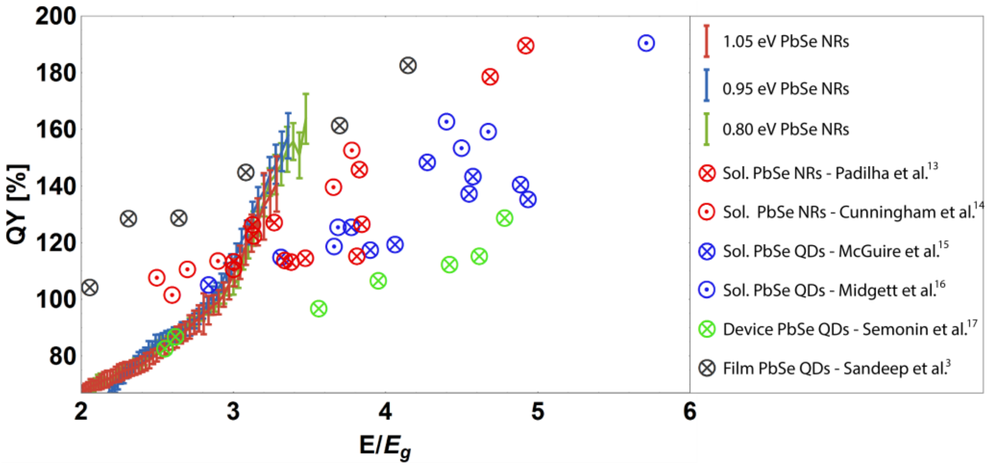

Supplementary Figure 11: Comparison of IQEs of PbSe NR devices (E_g = 1.05 eV, 0.95 eV and 0.80 eV) with MEG quantum yields of PbSe QDs in solution[11,12], PbSe QDs in films[3,13] and PbSe NRs in solution[14,15]. Error bars show the mean standard error of multiple independent solar cells (6 cells for 1.05 eV, 5 cells for 0.95 eV and 5 cells for 0.80 eV).

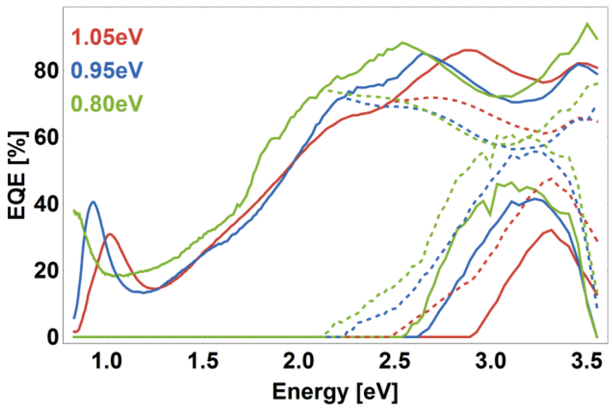

Supplementary Figure 12: Calculated EQEs used for determining the increase in photocurrent due to MEG. The solid and dashed lines indicate the EQE assuming MEG occurs with an IQE greater than 100 or 80 (5) respectively. The fraction of the IQE in excess of 100% or 80% is assumed to be due to MEG, and we weight the measured EQE by that fraction before integrating over the solar spectrum.

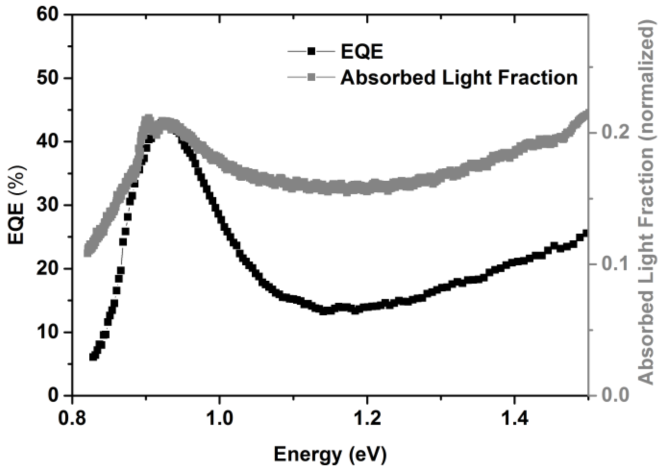

Supplementary Figure 13: EQE and absorbed light fraction of a film of QDs (E_g=0.95 eV). The dip in measured EQE after the first absorption peak is larger than expected from the fraction of light absorbed in the device assuming a constant IQE.

Supplementary Table 1: In order to reconstruct the short-circuit current from the individual EQEs we multiply the spectrally resolved photocurrent at each wavelength with the corresponding AM1.5G value and integrate over the entire spectrum. The respective error in % was taken from the error of each individual comparison between measured and calculated J_{SC}. We compared in total 4, 6 and 6 independent solar cells for NR devices of the bandgap 0.80 eV, 0.95 eV and 1.05 eV respectively.

E_g	Measured J_{SC}[mA/cm^2]	Calculated J_{SC}[mA/cm^2]	Error [%]
**1.05**	20.3 ± 1.1	20.2 ± 1.0	0.4
**0.95**	21.2 ± 1.1	21.2 ± 1.0	1.1
**0.80**	23.2 ± 0.6	22.6 ± 0.4	0.9

Supplementary Table 2: Calculated MEG contribution to the short-circuit current.[a] MEG taken as an IQE greater than 100 %. [b] MEG taken as an IQE greater than 80%.

E_g [eV]	Current attributed to MEG [mA/cm^2]	Current without MEG [mA/cm^2]	Enhancement due to MEG [%]
**1.05**[a]	0.3	20.4	1.7
**0.95**[a]	0.9	20.4	4.5
**0.80**[a]	1.3	23.2	5.8
**1.05**[b]	1.0	19.7	5.0
**0.95**[b]	2.0	19.4	10.2
**0.80**[b]	2.7	21.9	12.5

Supplementary Note 1: Optimisation of the nanorod synthesis

We optimised existing PbSe NR synthesis methods such that the NR aspect ratio was ca. 7 and the quantity of residual spherical quantum dots and hook-like structures is minimized. Recently, it has been demonstrated by Boercker *et al.* that both water and oleic acid (OA) influence the nanorod shape greatly.[1] For instance, while an increased water content reduces the aspect ratio of the final NRs, it was shown that excess quantities of uncoordinated OA in the Pb-oleate precursor solution promotes the formation of branched nanostructures. It has been discussed that a resulting increased reactivity of potential side reactions such as the reaction of tris(dimethylamino)phosphine (TDP) to bis(diethylamido)phosphorous acid (BDPA) are likely to drive these structural dependences on the content of free OA and water.

In order to reduce the branching ratio of the nanocrystals we explore the influence of different Pb to OA ratios (see Supplementary Figure 3) while keeping the water content at a minimum (fully degassed reactants). We find that at a stoichiometric ratio of 2.5 (OA:Pb) in the Pb-precursor solution produces the highest relative quantity of rod structures and keeps the unwanted population of branched nanostructures to a minimum. We note that with higher OA:Pb ratios the relative quantity of 0-dimensional quantum dots decreases further. As these crystals can be separated from the NRs via size-selective precipitation we evaluate an OA:Pb ratio of 2.5 as optimised stoichiometry for our Pb-precursor. In Supplementary Figure 3(b-d) we show additional transmission electron microscopy (TEM) images for the different OA:Pb ratios.

Supplementary References:

(1) Boercker, J. E.; Foos, E. E.; Placencia, D.; Tischler, J. G. *J. Am. Chem. Soc.* **2013**, *135*, 15071–15076.

(2) Ehrler, B.; Walker, B. J.; Böhm, M. L.; Wilson, M. W. B.; Vaynzof, Y.; Friend, R. H.; Greenham, N. C. *Nat. Commun.* **2012**, *3*, 1019.

(3) Sandeep, C. S. S.; ten Cate, S.; Schins, J. M.; Savenije, T. J.; Liu, Y.; Law, M.; Kinge, S.; Houtepen, A. J.; Siebbeles, L. D. a. *Nat. Commun.* **2013**, *4*, 2360.

(4) Gao, Y.; Aerts, M.; Sandeep, C. S. S.; Talgorn, E.; Savenije, T. J.; Kinge, S.; Siebbeles, L. D. a; Houtepen, A. J. *ACS Nano* **2012**, *6*, 9606–9614.

(5) Look, D.; Reynolds, D.; Sizelove, J. *Solid State Commun.* **1998**, *105*, 399–401.

(6) Yagi, E.; Hasiguti, R.; Aono, M. *Phys. Rev. B* **1996**, *54*, 7945–7956.

(7) Willis, R.; Olson, C.; O'Regan, B. *J. Phys. Chem. B* **2002**, *106*, 7605–7613.

(8) Baker, J. L.; Widmer-Cooper, A.; Toney, M. F.; Geissler, P. L.; Alivisatos, a P. *Nano Lett.* **2010**, *10*, 195–201.

(9) Rivest, J. B.; Swisher, S. L.; Fong, L.-K.; Zheng, H.; Alivisatos, a P. *ACS Nano* **2011**, *5*, 3811–3816.

(10) Ip, A. H.; Thon, S. M.; Hoogland, S.; Voznyy, O.; Zhitomirsky, D.; Debnath, R.; Levina, L.; Rollny, L. R.; Carey, G. H.; Fischer, A.; Kemp, K. W.; Kramer, I. J.; Ning, Z.; Labelle, A. J.; Chou, K. W.; Amassian, A.; Sargent, E. H. *Nat. Nanotechnol.* **2012**, *7*, 577–582.

(11) McGuire, J. A.; Joo, J.; Pietryga, J. M.; Schaller, R. D.; Klimov, V. I. *Acc. Chem. Res.* **2008**, *41*, 1810–1819.

(12) Midgett, A. G.; Hillhouse, H. W.; Hughes, B. K.; Nozik, A. J.; Beard, M. C. *J Phys. Chem. C* **2010**, 17486–17500.

(13) Semonin, O. E.; Luther, J. M.; Choi, S.; Chen, H.-Y.; Gao, J.; Nozik, A. J.; Beard, M. C. *Science* **2011**, *334*, 1530–1533.

(14) Cunningham, P. D.; Boercker, J. E.; Foos, E. E.; Lumb, M. P.; Smith, A. R.; Tischler, J. G.; Melinger, J. S. *Nano Lett.* **2013**, *13*, 3003–3003.

(15) Padilha, L. A.; Stewart, J. T.; Sandberg, R. L.; Bae, W. K.; Koh, W. K.; Pietryga, J. M.; Klimov, V. I. *Nano Lett.* **2013**, *13*, 1092–1099.
